# DCLA-Net: dual-encoder cross-level alignment network for ultrasound-based ectopic pregnancy mass segmentation

**DOI:** 10.3389/fphys.2026.1874479

**Published:** 2026-08-14

**Authors:** Yan Cheng, Xiaokang Ding, Weiwei Zhu

**Affiliations:** 1 Department of Obstetrics, Quzhou People’s Hospital, The Quzhou Affiliated Hospital, Wenzhou Medical University, Quzhou, China; 2 College of Mechanical Engineering, Quzhou University, Quzhou, China

**Keywords:** dual-attention fusion module, dual-encoder architecture, ectopic pregnancy mass segmentation, multi-scale context-aware fusion module, transformer

## Abstract

**Introduction:**

Benefiting from its real-time imaging capability and non-invasive nature, ultrasound imaging has become an important modality for the diagnosis of ectopic pregnancy. However, the segmentation of ectopic pregnancy masses in ultrasound images remains a challenging task due to low image contrast, indistinct boundaries, severe noise, and substantial variations in lesion size and shape. To tackle these issues, we propose DCLA-Net, a dual-encoder cross-level alignment network for ectopic pregnancy mass segmentation.

**Methods:**

Specifically, DCLA-Net adopts a dual-encoder architecture composed of a convolutional neural network (CNN) branch and a Transformer branch, which are designed to capture local details and global contextual information. To enhance the integration of features from different semantic levels, a multi-scale context-aware fusion module (MSCAFM) is introduced to strengthen feature interaction and alignment across hierarchical representations. In addition, cross-level connections are established between the encoder and decoder to promote information propagation and alleviate the loss of fine details during feature transmission. Furthermore, a dual-attention fusion module (DAFM) is incorporated into the decoding stage to adaptively integrate channel-wise and spatial features, thereby improving feature representation capability.

**Results and Discussion:**

To evaluate the effectiveness of the proposed method, extensive experiments were conducted on a clinical Ectopic Pregnancy Mass dataset and a publicly available Carotid dataset. On the Ectopic Pregnancy Mass dataset, DCLA-Net achieved Dice of 0.8455, Mcc of 0.8425, and Jaccard of 0.7341. On the Carotid dataset, it obtained Dice of 0.9597, Mcc of 0.9588, and Jaccard of 0.9227. Compared with state-of-the-art methods, DCLA-Net consistently achieves superior performance across all evaluation metrics on both datasets. In addition, ablation studies were performed to further validate the effectiveness of each proposed component. The results demonstrate that the dual-encoder architecture, MSCAFM, and DAFM all contribute positively to the overall segmentation performance.

## 1 Introduction

Ectopic pregnancy is a common and potentially life-threatening gynecological condition in which a fertilized ovum implants outside the uterine cavity, most commonly in the fallopian tube. In recent years, its incidence and detection rate have shown an increasing trend with the wider use of assisted reproductive technologies and improved early pregnancy screening. Among its imaging manifestations, ectopic pregnancy masses are of particular clinical significance, as they reflect abnormal gestational implantation and related pathological changes. However, this condition is highly variable in morphology and progression. Some lesions remain relatively stable, whereas others may enlarge or rupture rapidly, leading to severe intra-abdominal bleeding and even life-threatening complications. Clinically, early symptoms are often nonspecific or absent, while progressive cases may present with abdominal pain, vaginal bleeding, dizziness, or even hemorrhagic shock. Therefore, early identification and accurate assessment of ectopic pregnancy masses are essential for timely diagnosis and treatment.

At present, ultrasound imaging has become the primary modality for the detection and clinical management of ectopic pregnancy due to its real-time capability, non-invasive nature, and wide accessibility (Zhang et al., 2025a). Clinical studies have shown that ultrasound not only provides essential information for identifying ectopic pregnancy masses but also plays a crucial role in monitoring lesion progression, guiding treatment decisions, and evaluating therapeutic outcomes. Therefore, accurate segmentation of ectopic pregnancy masses is of great importance for improving diagnostic consistency and supporting reliable quantitative analysis. Traditionally, the delineation of ectopic pregnancy masses in ultrasound images relies heavily on manual annotation by clinicians. However, this process is labor-intensive, time-consuming, and subject to inter-observer variability due to differences in experience and interpretation. In the early development of segmentation methods, conventional image processing techniques, such as thresholding (Hempstead et al., 2025), region growing (Chen et al., 2003), and model-based approaches (Fang et al., 2020), were widely explored. While these methods may perform adequately under relatively simple imaging conditions, they often struggle to achieve robust and accurate segmentation in ultrasound images. In addition, traditional approaches typically depend on handcrafted features and prior assumptions, which limits their adaptability and generalization ability in diverse clinical scenarios.

In recent years, deep learning-based methods (Venkatesh et al., 2026; Ren et al., 2026) have significantly improved the performance of medical image segmentation, especially in ultrasound image analysis. CNN-based architectures have shown great success due to their ability to learn hierarchical feature representations and capture local contextual information. However, CNNs are limited by their reliance on local receptive fields, which makes it challenging to model long-range dependencies and global context. To address these limitations, Transformer-based models have emerged as a promising solution for medical image segmentation. By utilizing self-attention mechanisms, Transformers can effectively capture long-range dependencies and model global context. Despite their strengths, Transformer-based methods struggle to preserve fine-grained local details, which are essential for precise boundary delineation (Gu et al., 2023). Furthermore, many existing approaches still rely on single-encoder architectures, which fail to fully leverage the complementary strengths of CNNs and Transformers. Additionally, the lack of sufficient cross-level feature interaction and alignment between the encoder and decoder stages further hampers segmentation accuracy.

To address these challenges, we propose DCLA-Net, a dual-encoder cross-level alignment network for ectopic pregnancy mass segmentation. The proposed framework combines a CNN encoder with a Transformer encoder to jointly exploit fine-grained local features and long-range contextual information. In addition, cross-level feature alignment and attention-based feature fusion are employed to enhance feature representation and improve segmentation accuracy in challenging ultrasound images. The main innovations of DCLA-Net are as:

1. A novel dual-encoder cross-level alignment network is proposed for ectopic pregnancy mass segmentation in ultrasound images. By integrating a CNN branch and a Transformer branch within a unified framework, the proposed model effectively captures both fine-grained local details and long-range global contextual information, thereby addressing the limitations of conventional single-encoder architectures.
2. 2A multi-scale context-aware fusion module is designed to enhance hierarchical feature interaction and integration. This module enables effective aggregation of multi-level features across different semantic stages, improves contextual representation, and alleviates information loss, thereby facilitating more accurate lesion localization and structural consistency.
3. A dual-attention fusion module is introduced to refine feature fusion in the decoding stage. By adaptively integrating channel-wise and spatial information, DAFM enhances feature discrimination and enables more precise boundary delineation under complex ultrasound imaging conditions.

## 2 Related work

### 2.1 U-Net and its variants

The U-shaped encoder-decoder architecture introduced by Ronneberger et al (Ronneberger et al., 2015). has become one of the most influential architectures for medical image segmentation. Owing to its symmetric encoder-decoder structure and skip connections, U-Net effectively combines low-level spatial information with high-level semantic representations, making it particularly suitable for ultrasound image segmentation. Based on this architecture, numerous variants have been developed to address the challenges posed by complex ultrasound images, such as blurred boundaries, low contrast, and heterogeneous lesion appearance. For instance (Ganne and Balakrishna, 2026), proposed a neural architecture search strategy to automatically identify optimal building blocks within the encoder-decoder framework, using optimization techniques to explore model configurations and select efficient components for improved performance and efficiency. Abdel-Nabi (Abdel-Nabi, 2026) proposed a scale-aligned feature excitation module to effectively coordinate multi-resolution features. By enhancing the interaction among features at different scales, this module improves contextual consistency and strengthens the representation of semantic information (Chen et al., 2026). introduced a dual-branch attention gating mechanism into the skip connections to enable adaptive feature weighting. By selectively emphasizing informative features and suppressing irrelevant responses, this design enhances cross-scale contextual integration and improves feature fusion (Jiang et al., 2025). proposed a multi-scale linear attention gating mechanism to refine skip connections. This design suppresses redundant feature responses while preserving informative cues, thereby improving decoding efficiency and promoting more effective feature reconstruction (Li and Lin, 2025). integrated a multi-scale residual attention module into the encoder to enhance feature extraction. By combining residual learning with attention mechanisms across multiple scales, this design improves feature representation and strengthens the model’s ability to capture complex patterns (Zhu et al., 2025). developed a multi-scale attention module combined with a boundary supplementation module, which enhances feature extraction at different scales and compensates for boundary information loss. Although CNN-based architectures have achieved remarkable success, their convolutional operations mainly focus on local receptive fields, making it difficult to capture long-range dependencies and global contextual information. To overcome this limitation, CNN-Transformer hybrid architectures have attracted increasing attention by integrating the complementary strengths of convolutional networks and self-attention mechanisms. For instance (Ovi et al., 2026), proposed an uncertainty-enhanced pyramid vision transformer to improve feature representation by incorporating uncertainty modeling into multi-scale Transformer architectures (Wu et al., 2026). proposed an iterative prototype detail-enhanced transformer that employs a cyclic refinement process with bias correction and detail enhancement to progressively improve feature representation (Wang et al., 2026). introduced a spatial-channel cross-transformer block with skip connections to enhance feature integration across spatial and channel dimensions (Pang et al., 2025). adopted an adaptive perception transformer to capture both fine-grained local details and broader contextual information from the background (Wang et al., 2025). proposed a three-level spatiotemporal transformer to extract and fuse features from spatial, temporal, and depth dimensions (Pallawi and Singh, 2026). introduced a locally enhanced feedforward vision transformer, which improves feature extraction by effectively capturing spatially relevant information in the input data (Garbaz et al., 2026). proposed a transformative hierarchical context integration transformer that enhances feature aggregation by improving contextual understanding across multiple levels (Yin and Xu, 2026). developed a wavelet-Fourier hybrid approach that leverages complementary frequency-domain representations to capture complex multi-scale and multi-directional relationships.

### 2.2 Dual-branch architectures

Dual-branch architectures have been widely explored in medical image segmentation due to their ability to learn complementary feature representations through parallel pathways. Unlike single-branch frameworks, these architectures introduce two cooperative branches in the encoder, decoder, or both, so as to better capture diverse characteristics of medical images and enhance segmentation performance. For example (Wang et al., 2024), developed a dual-encoder architecture combining a CNN branch and a graph neural network branch. By jointly leveraging local feature extraction and graph-based relational modeling, the proposed method improves the ability to capture both spatial and structural information (Gao et al., 2026). integrated a CNN branch for local feature extraction with a vision transformer branch for modeling long-range dependencies (Chen et al., 2025). introduced a dual-branch encoder architecture consisting of a visual state space encoder and a residual network branch. The former is designed to model global dependencies, whereas the latter focuses on learning hierarchical local feature representations (El-Shafai et al., 2025). developed a dual-branch framework combining a boundary-sensitive U-Net with a segment anything model branch, allowing the model to jointly refine edge information and benefit from large-scale pretrained segmentation knowledge (Li et al., 2025). developed a dual-branch encoder combining the SE-U-Net for local feature extraction with a Transformer for global context modeling, thus enhancing the model’s ability to capture multi-scale features (Ren et al., 2025). developed a dual-branch parallel encoding network that integrates CNN with a visual state space model, allowing the model to capture both fine-grained local features and long-range contextual dependencies (Qi et al., 2024). introduced a dual-branch encoder architecture with a residual decoder, where both encoders work synergistically within the shared decoder to improve feature fusion and enhance segmentation performance.

## 3 Methods

### 3.1 Network architecture of DCLA-Net

**Figure 1:**
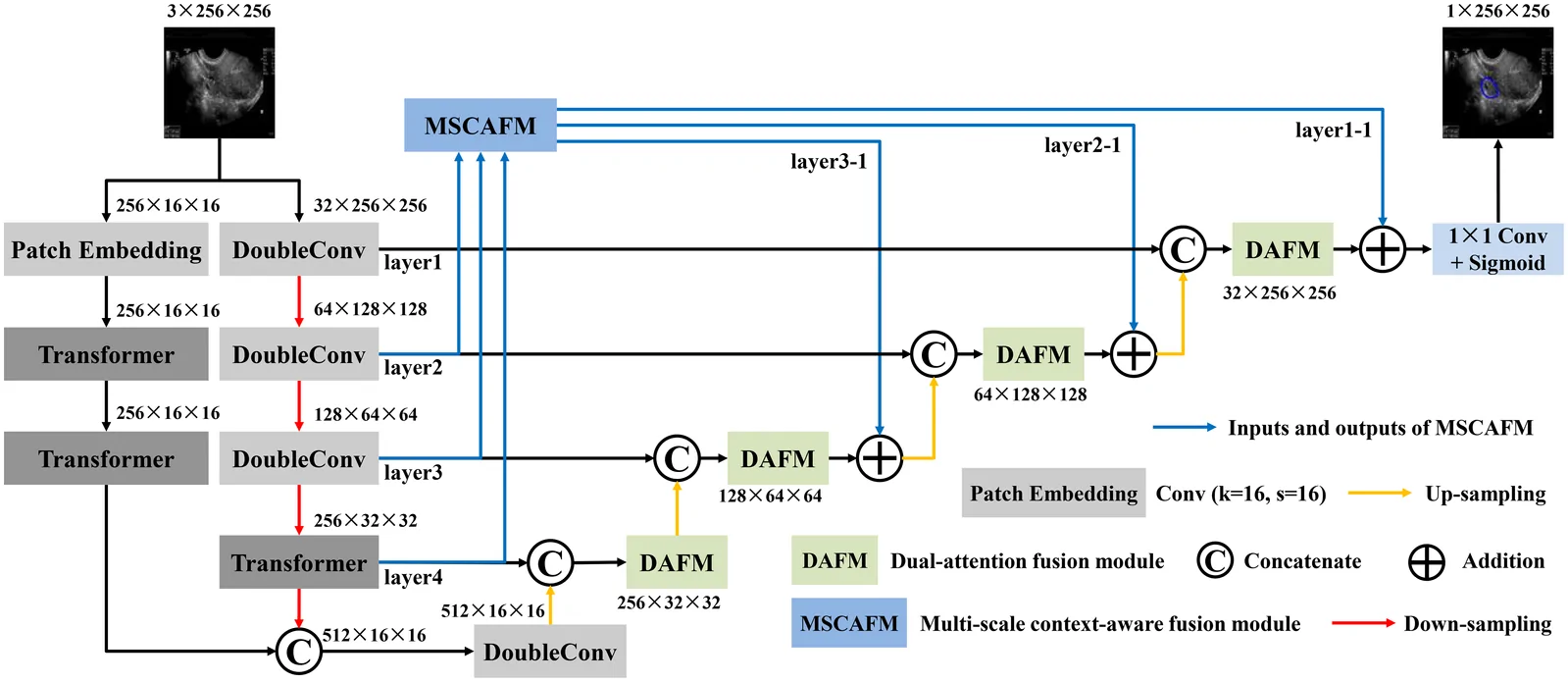
Network architecture of DCLA-Net.

Despite the success of U-Net in medical image segmentation, its application to ultrasound image segmentation, particularly for ectopic pregnancy mass segmentation, still faces several challenges. The standard convolutional layers in the encoder are often insufficient for capturing complex multi-scale features, especially when lesions exhibit substantial variations in shape, size, and boundary appearance. Moreover, the conventional skip connections in U-Net typically rely on direct concatenation between encoder and decoder features, which cannot effectively bridge the semantic gap across different feature levels and therefore limits the full exploitation of complementary information. To address these issues, we propose DCLA-Net, a dual-encoder cross-level alignment network for ultrasound-based ectopic pregnancy mass segmentation, as illustrated in **Figure 1**. Specifically, the input ultrasound image is simultaneously fed into two parallel encoding branches. In the left Transformer branch, the input is first processed by a patch embedding operation with a kernel size of 16 and a stride of 16, which transforms the image into a sequence of patch tokens. These tokens are then passed through stacked Transformer blocks to model long-range dependencies and capture global contextual information. In the right CNN branch, the first three stages are composed of DoubleConv blocks, each consisting of two consecutive convolutional layers, with progressive down-sampling, as indicated by the red arrows in **Figure 1**, while the fourth stage is built upon a Transformer. The feature maps generated at different stages are denoted as layer1, layer2, layer3, and layer4, corresponding to different semantic levels. To strengthen the interaction among features at different semantic levels, a multi-scale context-aware fusion module is introduced. This module extracts the features from the second to the fourth layers of the CNN branch, aggregates them, and then adds the resulting features to the first to the third layers of the decoder. By modeling cross-level dependencies, the module promotes effective information exchange across different scales and alleviates the semantic discrepancy between shallow and deep features. In addition, the conventional DoubleConv blocks in the first to the fourth decoder layers are replaced with the dual-attention fusion module, thereby significantly enhancing the model’s ability to capture both fine local details and global contextual information. Finally, the fused features are passed through a 1×1 convolution followed by a Sigmoid activation to generate the final segmentation map, which is visualized as a blue-colored mask in **Figure 1** for clarity.

### 3.2 Transformer module

**Figure 2:**
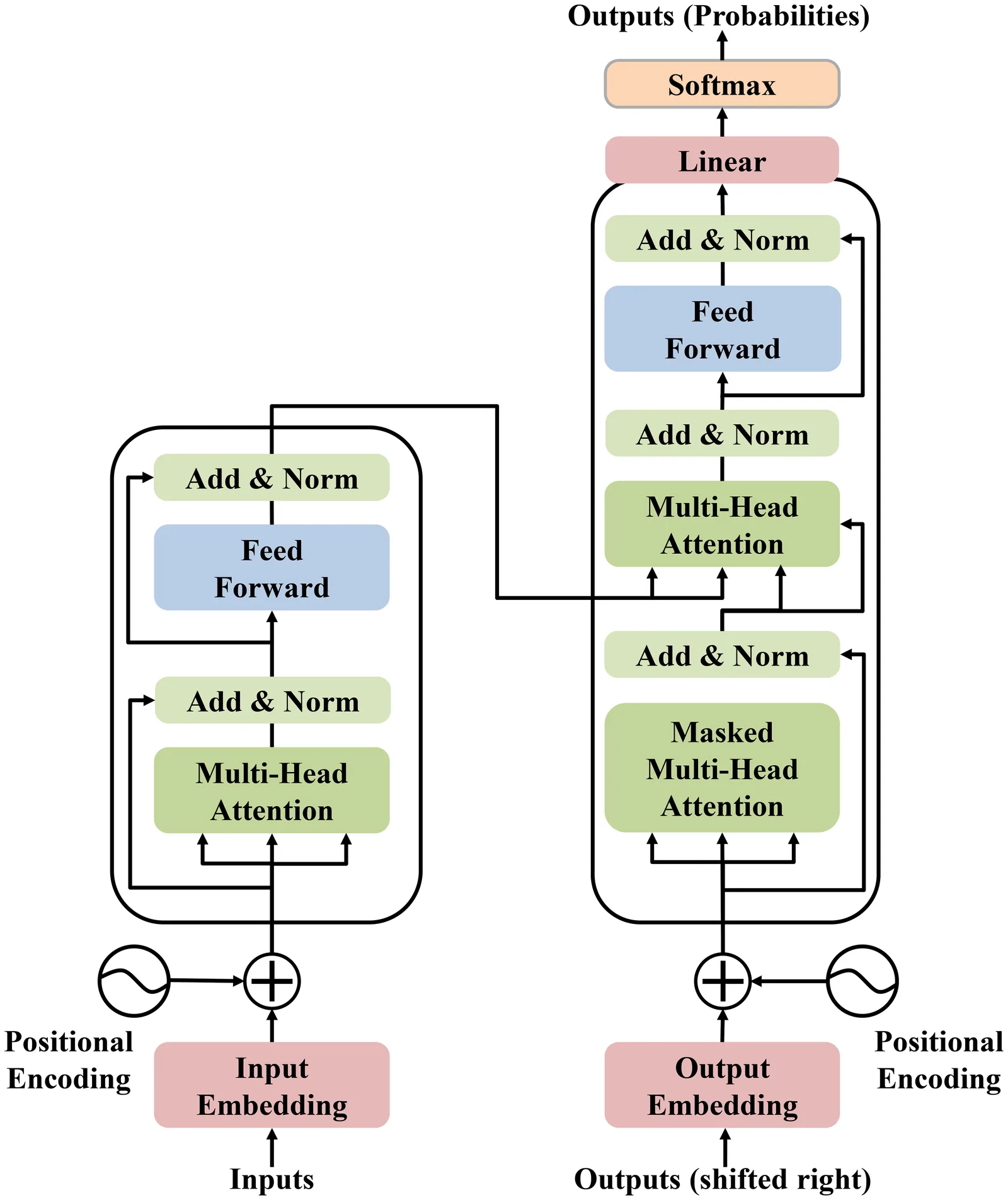
Structure of transformer module.

The Transformer is an attention-based deep learning model originally proposed by (Vaswani et al., 2017), as shown in **Figure 2**. In DCLA-Net, only the encoder component of the original Transformer architecture is adopted, which is implemented using PyTorch’s nn.TransformerEncoderLayer and nn.TransformerEncoder. Firstly, the input features are transformed into token representations through an embedding operation, and positional encoding is added to preserve spatial order information. These tokens are then fed into several stacked Transformer encoder layers. Each encoder layer mainly consists of a multi-head self-attention module and a feed-forward network. The multi-head self-attention module establishes relationships among all tokens, enabling the network to capture long-range dependencies and global contextual cues. Subsequently, residual connections and layer normalization are applied to stabilize feature learning. The feed-forward network further transforms and enhances the token features, followed again by residual addition and normalization. Through stacked encoder layers, the Transformer progressively strengthens global semantic representation and generates context-enhanced features, which provide complementary information for subsequent feature fusion. In the dual-encoder architecture of DCLA-Net, Transformer modules are introduced into the second and third stages of the left branch and the fourth stage of the right branch. This multi-stage design progressively incorporates global contextual information into hierarchical feature representations while preserving the complementary characteristics of the CNN and Transformer branches, thereby facilitating subsequent feature fusion in the decoder.

### 3.3 Multi-scale context-aware fusion module

**Figure 3:**
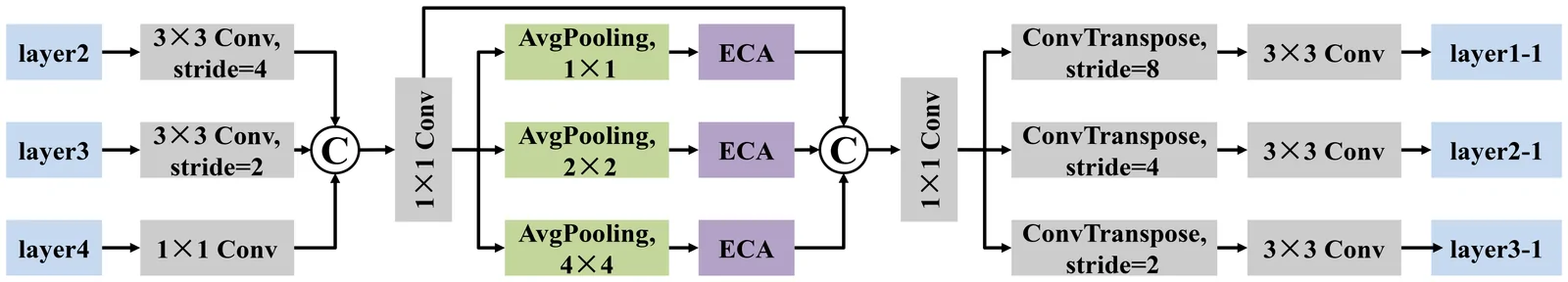
Structure of multi-scale context-aware fusion module.

To enhance cross-level feature interaction and effectively capture contextual information at multiple scales, a multi-scale context-aware fusion module is proposed, as illustrated in **Figure 3**. This module aims to aggregate features from different semantic levels and generate context-enhanced representations for subsequent decoding. Specifically, the input to MSCAFM consists of feature maps from layer2, layer3, and layer4. To align their spatial resolutions, the feature maps from layer2 and layer3 are first processed using 3×3 convolutions with strides of 4 and 2, while the feature map from layer4 is adjusted using a 1×1 convolution. These processed features are then concatenated and further transformed by a 1×1 convolution to unify channel dimensions and fuse multi-level information. To capture contextual information at different receptive fields, the fused feature is fed into three parallel branches with different pooling scales, including 1×1, 2×2, and 4×4 average pooling. Each pooled feature is subsequently refined by an efficient channel attention (ECA) (Wang et al., 2020) module to adaptively recalibrate channel-wise responses. This design enables the model to emphasize informative features while suppressing less relevant ones under different contextual scales. The outputs of these branches are then concatenated and passed through another 1×1 convolution to further integrate multi-scale contextual information. Finally, to match the spatial resolutions required by the decoder, the fused feature is progressively upsampled through a series of transpose convolution (ConvTranspose) layers with strides of 8, 4, and 2, respectively. Each upsampled feature is followed by a 3×3 convolution to refine spatial details, producing the outputs layer1-1, layer2-1, and layer3-1, which are used for subsequent feature fusion in the decoding stage. Overall, the proposed MSCAFM effectively integrates multi-level features and captures rich contextual information across different scales. By combining multi-scale pooling, channel attention, and hierarchical feature alignment, the module significantly reduces the semantic gap between features of different levels and improves the representation capability of the network.

**Figure 4:**
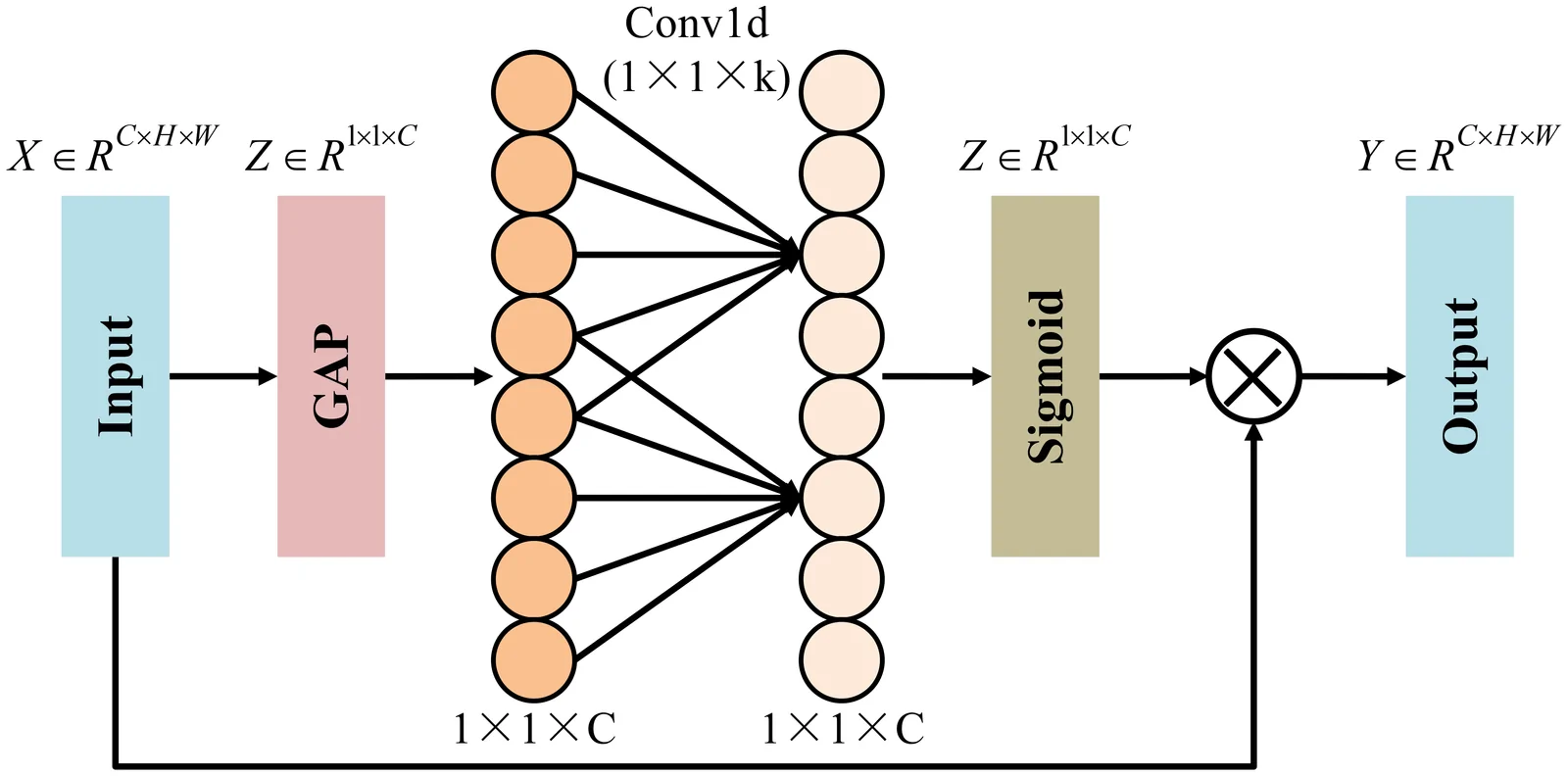
Structure of efficient channel attention module.

To further clarify the channel recalibration process, the structure of the ECA module used in MSCAFM is illustrated in **Figure 4**. For an input feature map $X\in R^{C\times H\times W}$, global average pooling (GAP) is first used to aggregate spatial information from each channel, producing a compact channel descriptor $Z\in R^{1\times 1\times C}$. This descriptor provides a global response representation for all channels. Instead of using fully connected layers for channel recalibration, ECA employs a lightweight one-dimensional convolution to capture local dependencies among neighboring channels. The convolution operation models cross-channel interactions while avoiding dimensionality reduction, thereby preserving useful channel information and reducing parameter overhead. After that, a sigmoid function is applied to generate channel attention weights. These weights are then multiplied with the original input feature map to obtain the recalibrated output feature. The entire process is defined in Equation 1:

(1) z=GAP(X);α=σ(Conv1D(z));Y=X⊗α

where Conv1D represents one-dimensional convolution, σ is the sigmoid activation function, ⊗ denotes channel-wise multiplication, and Y is the output feature map. By adaptively assigning different weights to different channels, the ECA module strengthens informative responses and suppresses less relevant features. Therefore, it can enhance feature representation in multi-scale fusion while maintaining a lightweight computational design.

### 3.4 Dual-attention fusion module

**Figure 5:**
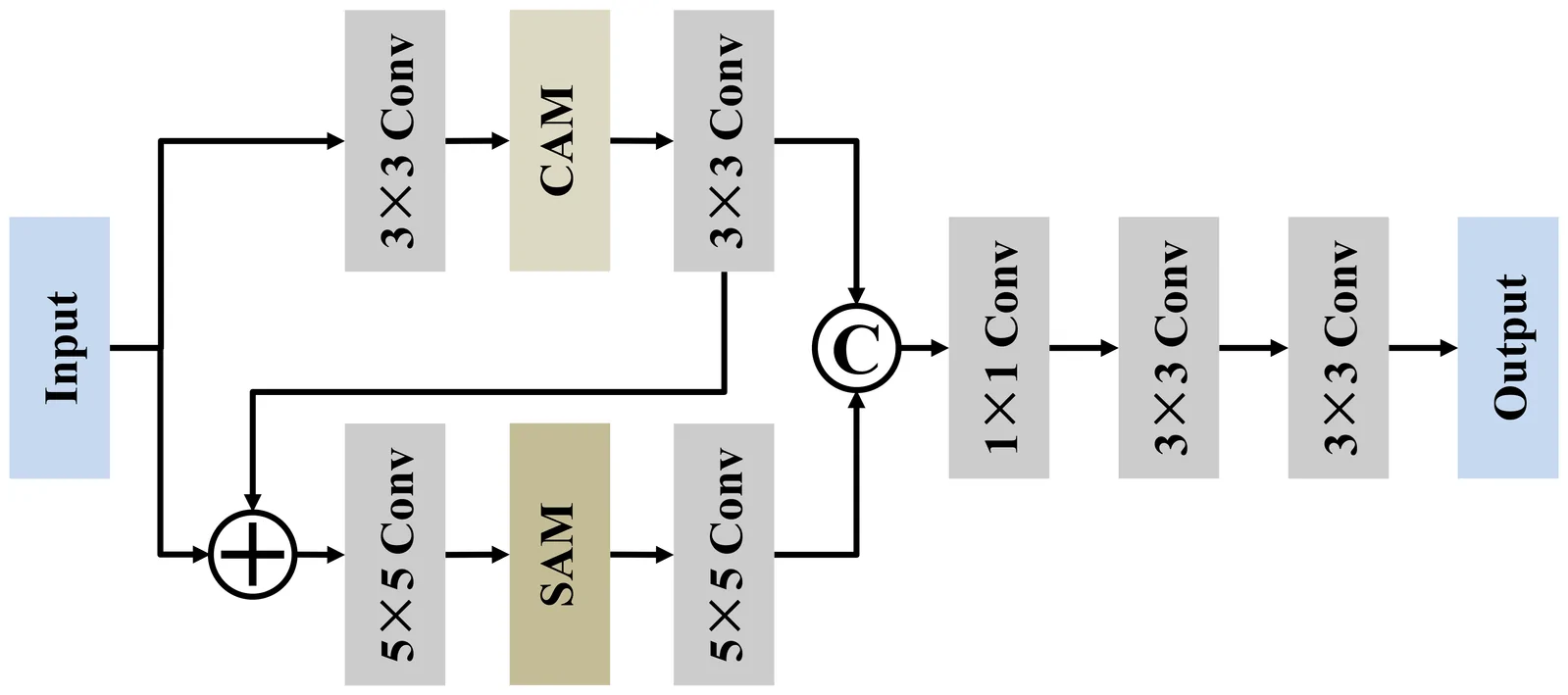
Structure of dual-attention fusion module.

In the decoding stage, we replace the traditional convolutional blocks with the dual-attention fusion module to improve the fusion of features, as shown in **Figure 5**. The DAFM enhances feature representation by leveraging both channel-wise and spatial attention mechanisms, which allows the network to focus on informative regions and suppress irrelevant background noise. Given an input feature map, DAFM first constructs two complementary branches. In the upper branch, the input feature is processed by a 3×3 convolution, followed by the channel attention module (CAM) and another 3×3 convolution, aiming to enhance channel-wise discriminative responses. Meanwhile, the output of the second 3×3 convolution in the upper branch is combined with the original input feature through element-wise addition, and the resulting feature is then fed into the lower branch. In the lower branch, a 5×5 convolution is first used to capture broader contextual information, followed by the spatial attention module (SAM) and another 5×5 convolution to refine spatially informative regions. The outputs of the upper and lower branches are then concatenated and passed through a 1×1 convolution to integrate channel information. Finally, two successive 3×3 convolutions are applied to further refine the fused representation and generate the output feature map. Through this design, DAFM effectively combines channel attention, spatial attention, local feature extraction, and broader contextual modeling, thereby improving feature fusion and enhancing segmentation accuracy.

**Figure 6:**
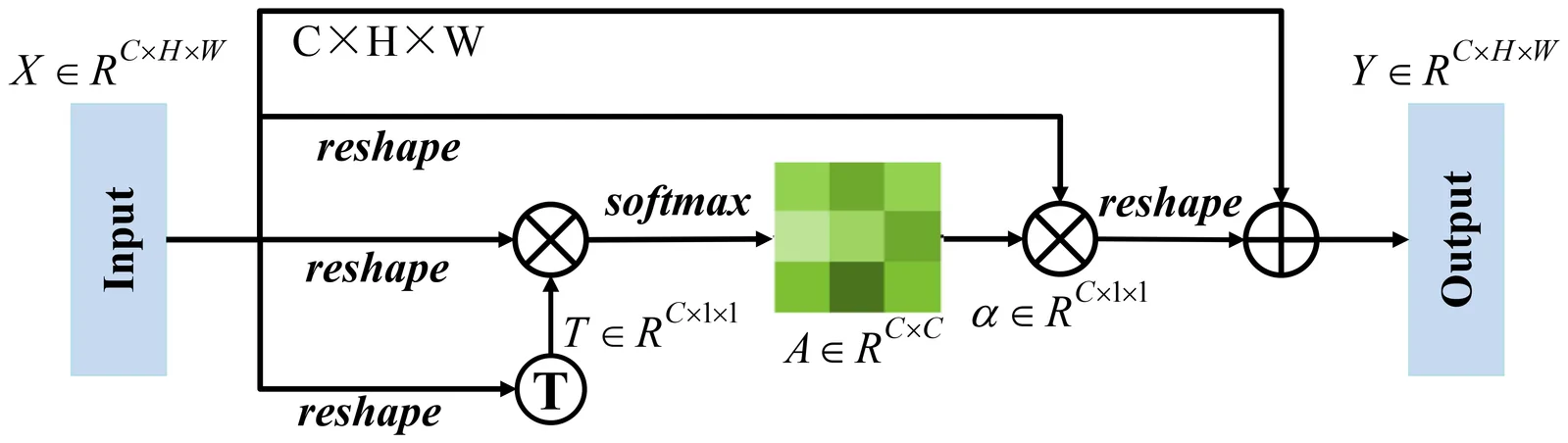
Structure of channel attention module.

The channel attention module (Ye et al., 2025) aims to strengthen informative channel responses by explicitly modeling the dependencies among different feature channels, as illustrated in **Figure 6**. Given an input feature map $X\in R^{C\times H\times W}$, the feature is first reshaped into two matrices for channel affinity computation. One reshaped feature is transposed to obtain $T\in R^{C\times 1\times 1}$, while the other is kept in its original reshaped form. Matrix multiplication is then performed between these two feature representations to generate a channel correlation matrix $A\in R^{C\times C}$, which describes the relationships among different channels. Subsequently, a softmax operation is applied to normalize the correlation matrix, producing the channel attention weights. These attention weights are multiplied with the reshaped feature representation to obtain the recalibrated feature $\alpha \in R^{C\times 1\times 1}$. Finally, the recalibrated feature is reshaped back to its original spatial dimension and combined with the input feature through a residual connection, yielding the output feature $Y\in R^{C\times H\times W}$. Through this process, CAM adaptively emphasizes informative channels while suppressing less relevant responses, thereby improving feature discrimination and enhancing the representation capability of the network.

**Figure 7:**
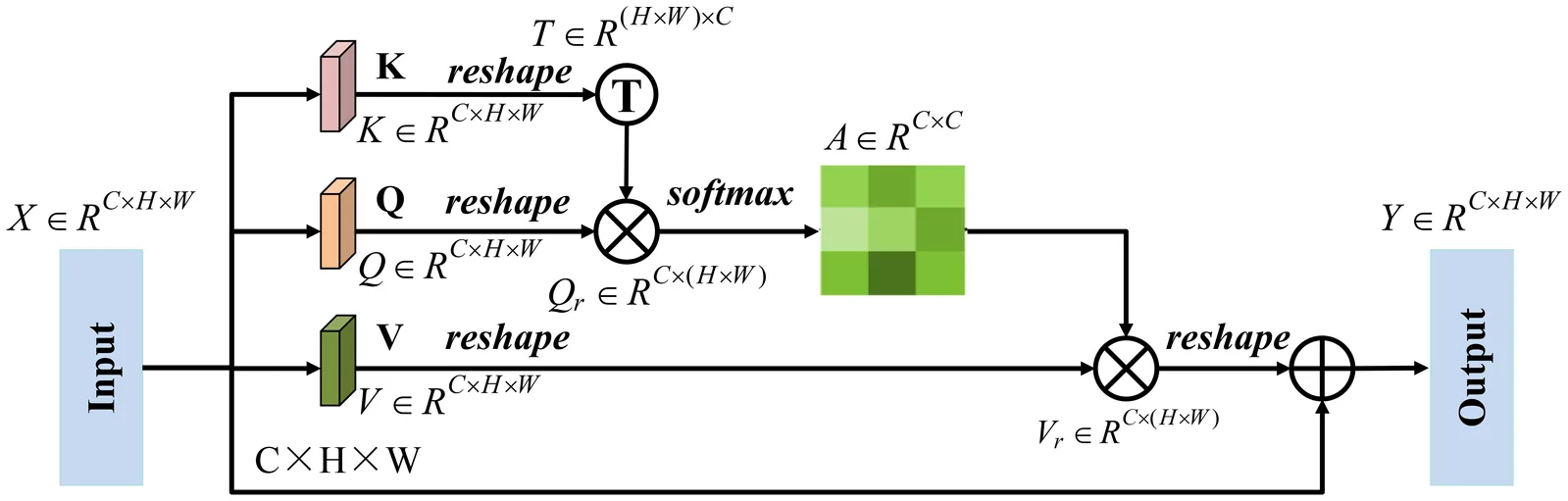
Structure of spatial attention module.

The spatial attention module (Hu et al., 2024) is designed to capture long-range spatial dependencies and enhance the representation of informative regions, as illustrated in **Figure 7**. Given an input feature map $X\in R^{C\times H\times W}$, three feature embeddings, namely query (Q), key (K), and value (V), are first generated through linear projections, where Q, K, and V have the same dimension as the input feature map. The query feature is reshaped into $Q_{r}\in R^{C\times (H\times W)}$, while the key feature is reshaped and transposed to obtain $T\in R^{(H\times W)\times C}$. Matrix multiplication is then performed between *Q_r_* and *T*, followed by a softmax operation, to generate the normalized attention matrix $A\in R^{C\times C}$, which describes the correlations among different feature channels. Subsequently, the value feature is reshaped into $V_{r}\in R^{C\times (H\times W)}$, the attention matrix A is multiplied with *V_r_* to produce an attention-enhanced feature representation. The resulting feature is then reshaped back to the original spatial dimension and combined with the input feature through an element-wise residual connection, yielding the output feature $Y\in R^{C\times H\times W}$. Through this process, SAM enables the model to emphasize relevant spatial regions while preserving the original feature information, thereby improving the ability to capture global spatial context and enhancing overall feature representation.

### 3.5 Loss function

For the task of ectopic pregnancy mass segmentation, we adopt the Dice loss (Luo et al., 2024; Yuan et al., 2024) function due to its effectiveness in addressing class imbalance and its sensitivity to small structures. The Dice coefficient is widely used in medical image segmentation tasks, as it directly measures the overlap between the predicted segmentation and the ground truth, providing a quantitative evaluation of segmentation accuracy. The Dice loss is defined in Equation 2:

(2) $$L_{Dice}=1-\frac{2\sum_{i=1}^{N}y_{i}{\hat{y}}_{i}}{\sum_{i=1}^{N}y_{i}^{2}+\sum_{i=1}^{N}{\hat{y}}_{i}^{2}}$$

where *y_i_* and ${\hat{y}}_{i}$ denote the predicted value and ground-truth label of the *i*-th pixel, N is the total number of pixels.

## 4 Experiments and discussion

### 4.1 Dataset

**Figure 8:**
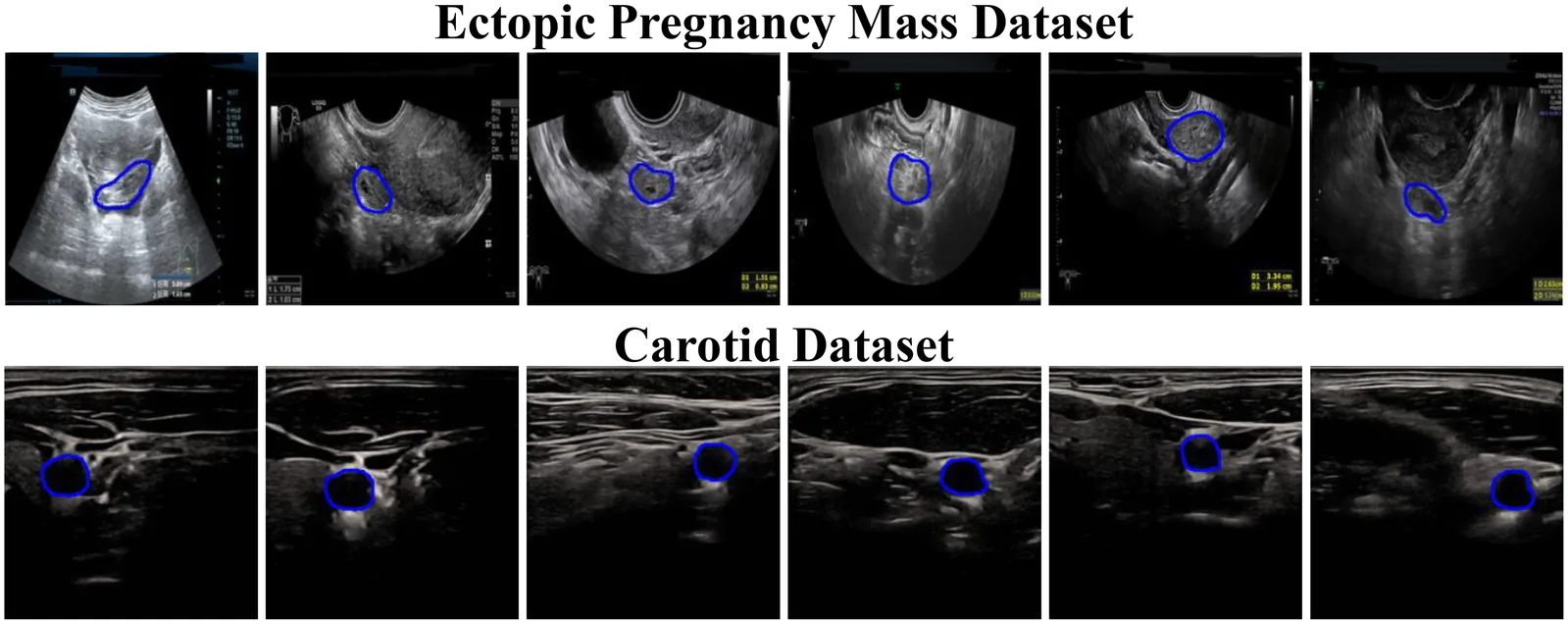
Representative examples from the Ectopic Pregnancy Mass and Carotid datasets. The top row shows images from the Ectopic Pregnancy Mass dataset, and the bottom row shows images from the Carotid dataset. The corresponding ground-truth annotations are outlined in blue.

To evaluate the performance of the proposed DCLA-Net, we conducted experiments on two distinct datasets: the self-constructed Ectopic Pregnancy Mass dataset from Quzhou People’s Hospital and the publicly available Carotid dataset (Momot, 2022). The Ectopic Pregnancy Mass dataset consists of real ultrasound images that were manually annotated by experienced clinicians to provide accurate lesion masks for supervised learning and performance evaluation. The dataset contains a total of 1,033 ultrasound images collected from 759 patients, with each patient contributing 1~3 images. To avoid patient-level data leakage and reduce the influence of highly correlated frames from the same examination, the dataset was partitioned at the patient level. Specifically, all images from the same patient were assigned exclusively to one subset, ensuring that no patient appeared in more than one of the training, validation, and testing sets. The resulting split comprised 621 images from 479 patients for training, 206 images from 143 patients for validation, and 206 images from 137 patients for testing. These images represent a wide range of ectopic pregnancy masses and capture the challenges associated with variations in lesion size, shape, appearance, and boundary characteristics. The publicly available Carotid dataset contains 1,100 ultrasound images acquired from 11 different subjects. Each subject underwent at least one examination of both the left and right carotid arteries. The images were manually annotated by technicians and subsequently verified by experts to generate the corresponding ground-truth segmentation masks. Since the publicly available dataset does not provide subject identifiers or subject-level grouping information, subject-wise partitioning was not feasible. Therefore, the images were randomly divided into training, validation, and testing sets with a ratio of 3:1:1, resulting in 660, 220, and 220 images. Representative examples from the Ectopic Pregnancy Mass and Carotid datasets are shown in **Figure 8**.

### 4.2 Evaluation metrics

To evaluate the segmentation performance of the proposed DCLA-Net in a quantitative manner, three commonly used metrics were employed: Dice (Wan et al., 2025a; Li et al., 2022), Matthews correlation coefficient (Mcc) (Wan et al., 2025b; Shang et al., 2024), and (Yang et al., 2024; Yeung et al., 2023). Among them, Dice measures the overlap between the predicted segmentation and the ground truth, Mcc evaluates the overall classification quality by considering true positives, true negatives, false positives, and false negatives, while Jaccard measures the intersection-over-union between the predicted and reference regions. For all three metrics, higher values indicate better segmentation performance. The Dice, Mcc, and Jaccard metrics are defined in Equations 3–5:

(3) $$Dice=\frac{2TP}{2TP+FN+FP}$$

(4) $$Mcc=\frac{TP\times TN-FP\times FN}{\sqrt{\left(TP+FN)(TP+FP)(TN+FN)(TN+FP\right)}}$$

(5) $$Jaccard=\frac{TP}{TP+FN+FP}$$

### 4.3 Implementation details

All experiments were conducted based on the PyTorch 2.0.0 platform. The model was trained and evaluated on a high-performance workstation equipped with an NVIDIA GeForce RTX 4090 GPU (24 GB memory). During training, the Adam optimizer was adopted to update network parameters, with the learning rate initialized at 1×10^-^³. The training procedure was carried out for a total of 200 epochs, and each mini-batch contained 16 samples. To ensure consistency in input representation, all images were preprocessed by resizing them to 256×256. The above configuration was uniformly applied in all experiments to guarantee a fair and reliable evaluation.

### 4.4 Ablation study

The ablation study was performed to verify the effectiveness of each component in DCLA-Net, as shown in **Table 1**. The ablation configurations were constructed progressively following the development of the proposed network. Specifically, the Baseline+Dual-encoder (DoubleConv only) configuration consists of two encoder branches built entirely with DoubleConv blocks, without introducing any Transformer modules. The convolution-based left encoder is subsequently redesigned using a patch embedding layer followed by Transformer blocks, resulting in the final hybrid CNN–Transformer dual-encoder architecture shown in **Figure 1**. This progressive design enables the contribution of each component to be evaluated independently. The baseline U-Net achieved Dice, Mcc, and Jaccard scores of 0.7806, 0.7769, and 0.6425. When the single encoder was replaced with the dual-encoder architecture without Transformer modules, the performance increased to 0.8273, 0.8241, and 0.7066. This indicates that the dual-branch encoding strategy can extract more complementary features and improve lesion representation even without introducing the Transformer module. After incorporating the Transformer modules into the dual-encoder architecture, Dice, Mcc, and Jaccard further improved to 0.8363, 0.8340, and 0.7213, demonstrating the benefit of global context modeling and long-range dependency learning. With the addition of MSCAFM, the model obtained higher scores of 0.8422, 0.8395, and 0.7296, suggesting that multi-scale contextual aggregation and cross-level feature interaction are effective for reducing semantic gaps. Finally, after introducing DAFM, the complete DCLA-Net achieved the best performance, with Dice, Mcc, and Jaccard scores of 0.8455, 0.8425, and 0.7341. To further demonstrate that the effectiveness of the proposed components is not limited to a single dataset, the same ablation experiments were also conducted on the public Carotid dataset. As shown in **Table 1**, similar performance trends are consistently observed. Replacing the single encoder with the dual-encoder architecture significantly improves the segmentation performance, while the incorporation of the Transformer module further enhances the Dice, MCC, and Jaccard scores. The introduction of MSCAFM and DAFM yields additional performance gains, with the complete DCLA-Net achieving the best overall performance (Dice = 0.9597, Mcc = 0.9588, and Jaccard = 0.9227). These consistent results across both datasets demonstrate that the proposed modules exhibit good generalizability rather than being specifically optimized for the Ectopic Pregnancy Mass dataset. To further evaluate the computational efficiency of the proposed network, three widely used complexity metrics, namely floating-point operations (GFLOPs), the number of trainable parameters (Params), and frames per second (FPS), are adopted. GFLOPs measure the computational cost required for a single forward inference, Params reflect the model size and memory requirement, while FPS indicates the inference speed during testing. Since the network architectures are identical on both datasets, the GFLOPs and parameter counts remain unchanged, while the measured FPS values are comparable. Therefore, the following discussion uses the Ectopic Pregnancy Mass dataset as a representative example. In terms of computational efficiency, the baseline U-Net requires 16.79 GFLOPs and 8.96M parameters, with an inference speed of 584.26 FPS. After introducing the dual-encoder structure, the parameter number increases to 12.70M and the speed decreases slightly to 533.41 FPS, while the computational cost remains relatively close at 17.75 GFLOPs. Notably, after incorporating the Transformer module, both the computational cost and the number of parameters decrease to 15.93 GFLOPs and 9.16M. This reduction is mainly attributed to the architectural redesign of the left encoder branch. Instead of using multiple DoubleConv blocks with progressive downsampling, the Transformer branch first applies a patch embedding layer with a kernel size and stride of 16, which directly reduces the input feature map from 256×256 to 16×16. Consequently, the subsequent Transformer blocks operate on substantially lower-resolution feature maps, significantly reducing both the computational cost and the number of trainable parameters compared with a fully convolutional encoder. Although the theoretical complexity is reduced, the inference speed decreases to 379.32 FPS because the self-attention operations, tensor reshaping, and matrix multiplications involved in the Transformer introduce additional runtime overhead that is not fully reflected by GFLOPs or parameter count. The introduction of MSCAFM substantially increases the model complexity, with the parameter count rising from 9.16M to 98.47M and GFLOPs increasing from 15.93 to 145.43, while the inference speed decreases to 268.47 FPS. Meanwhile, the Dice score improves from 0.8363 to 0.8422 (an absolute improvement of 0.0059). This increase in computational complexity mainly results from the multiple parallel multi-scale feature extraction branches and extensive feature aggregation operations within MSCAFM. After further incorporating DAFM, the complete DCLA-Net reaches 177.74 GFLOPs and 108.95M parameters, with an inference speed of 111.83 FPS, while achieving the highest segmentation accuracy among all ablation configurations. Overall, the experimental results indicate that the performance improvement of DCLA-Net is achieved at the expense of increased computational complexity. In particular, although MSCAFM provides measurable improvements in segmentation accuracy, it also contributes the largest increase in model complexity. Therefore, the proposed model represents a trade-off between segmentation accuracy and computational efficiency rather than an unconditional improvement in both aspects. Future work will focus on developing a lightweight version of MSCAFM by simplifying the multi-scale feature aggregation strategy and reducing redundant feature fusion operations, thereby improving computational efficiency while maintaining competitive segmentation performance.

**Table 1:** Ablation study on the ectopic pregnancy mass and carotid datasets.

Dateset	Method	Dice	Mcc	Jaccard	GFLOPs	Params(M)	FPS
Ectopic Pregnancy Mass	Baseline (U-Net)	0.7806	0.7769	0.6425	16.79	8.96	584.26
	Baseline+Dual-encoder (DoubleConv only)	0.8273	0.8241	0.7066	17.75	12.70	533.41
	Baseline+Dual-encoder+Transformer	0.8363	0.8340	0.7213	15.93	9.16	379.32
	Baseline+Dual-encoder+Transformer+MSCAFM	0.8422	0.8395	0.7296	145.43	98.47	268.47
	Baseline+Dual-encoder+Transformer+MSCAFM+DAFM	0.8455	0.8425	0.7341	177.74	108.95	111.83
Carotid	Baseline (U-Net)	0.9493	0.9484	0.9039	16.79	8.96	601.64
	Baseline+Dual-encoder (DoubleConv only)	0.9556	0.9547	0.9153	17.75	12.70	499.31
	Baseline+Dual-encoder+Transformer	0.9575	0.9565	0.9186	15.93	9.16	373.16
	Baseline+Dual-encoder+Transformer+MSCAFM	0.9581	0.9571	0.9200	145.43	98.47	233.11
	Baseline+Dual-encoder+Transformer+MSCAFM+DAFM	0.9597	0.9588	0.9227	177.74	108.95	111.51

**Figure 9:**
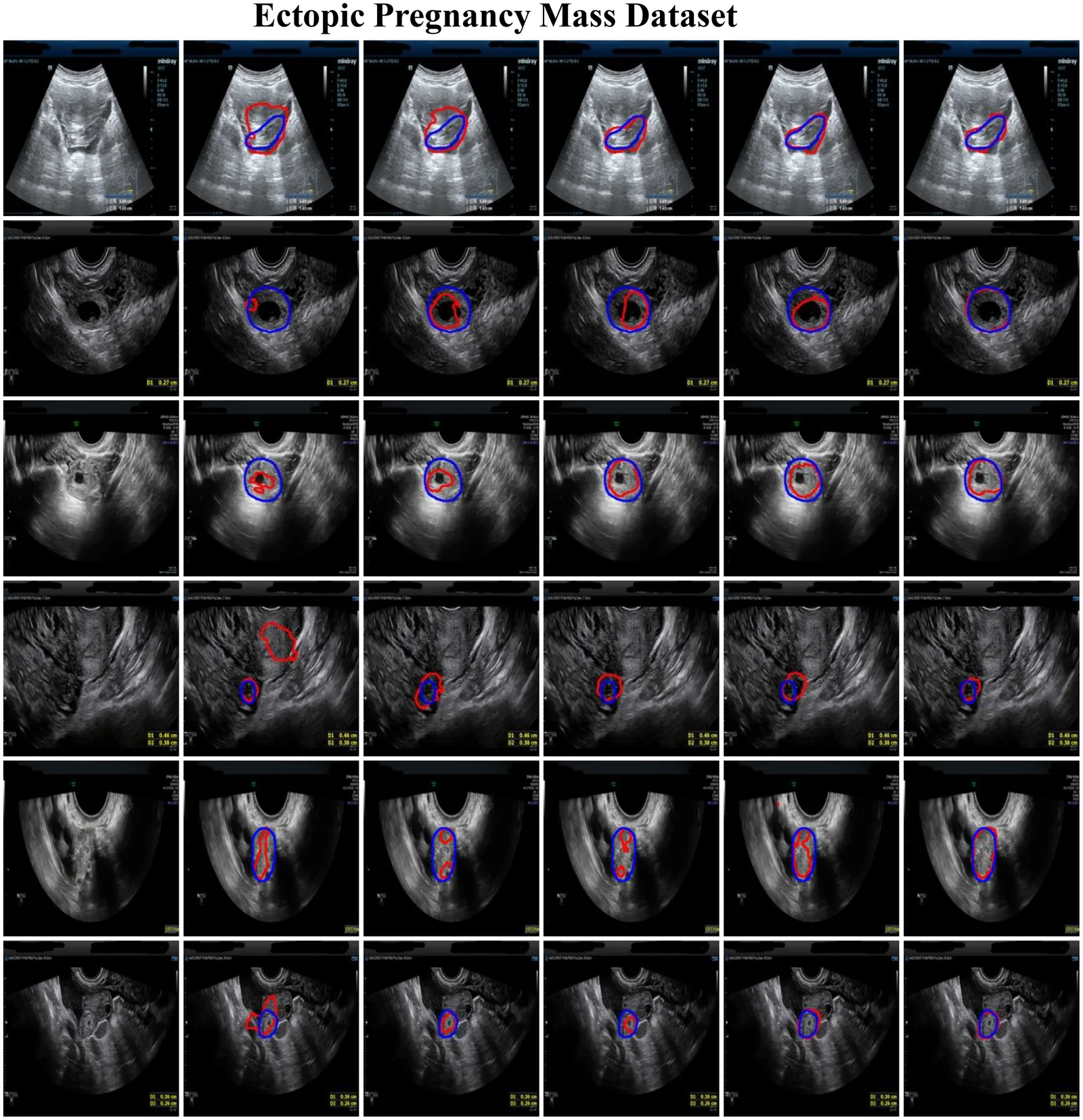
Qualitative comparison of the ablation study on the ectopic pregnancy mass dataset. The first column shows the original images, while the remaining columns display the results of Baseline, Baseline+Dual-encoder (DoubleConv only), Baseline+Dual-encoder+Transformer, Baseline+Dual-encoder+Transformer+MSCAFM, and Baseline+Dual-encoder+Transformer+MSCAFM+DAFM (DCLA-Net). The blue curves denote the ground truth annotations, and the red curves indicate the predicted masks.

As shown in **Figure 9**, the segmentation results are visualized by overlaying the ground-truth annotations (blue contours) and the predicted boundaries (red contours) on the original ultrasound images, providing a more intuitive comparison of the segmentation performance. The baseline U-Net tends to produce coarse predictions, with incomplete lesion regions and inaccurate boundaries, particularly in cases with low contrast or ambiguous structures. After introducing the dual-encoder design (DoubleConv only), the segmentation becomes more complete and stable, indicating enhanced feature extraction capability, although some boundary irregularities and false responses still exist. With the addition of the Transformer module, the predicted regions exhibit better structural consistency and closer alignment with the ground truth, reflecting the benefit of global context modeling. When MSCAFM is further integrated, the model shows improved localization and clearer lesion boundaries, suggesting that multi-scale feature fusion effectively reduces semantic discrepancies. Finally, after incorporating DAFM, the full DCLA-Net generates the most accurate results, with smoother contours and fewer spurious predictions. Overall, these observations confirm that each module contributes positively to segmentation performance, and their combination leads to more precise and robust lesion delineation.

### 4.5 Comparison study

**Table 2** presents a quantitative comparison between DCLA-Net and several representative segmentation methods on the Ectopic Pregnancy Mass dataset. The results are reported as mean ± standard deviation to provide a more comprehensive evaluation of segmentation performance and stability. Among the Transformer-based models, PVTFormer achieved the lowest performance, with Dice score of 0.6841, Mcc of 0.6794, and Jaccard of 0.5221. In comparison, TransRUPNet and TransResU-Net achieved relatively higher performance, with Dice scores of 0.7236 and 0.7488. These results indicate that Transformer-based models face greater challenges when applied to the Ectopic Pregnancy Mass dataset. Among the dual-branch models, DoubleU-Net achieved Dice, Mcc, and Jaccard scores of 0.7841, 0.7813, and 0.6466, while I2U-Net and DESENet obtained higher and comparable results, with Dice scores of 0.8258 and 0.8246. These results indicate that dual-branch feature extraction strategies can effectively enhance feature representation and achieve better segmentation performance on this dataset. The conventional encoder-decoder models, including A-Net, LMFR-Net, and GAC-Net, also demonstrated competitive performance, achieving Dice scores of 0.8104, 0.8022, and 0.8308, respectively. These improvements can be attributed to the incorporation of feature refinement strategies and attention mechanisms. In contrast, DCLA-Net achieved the highest numerical performance, with Dice, Mcc, and Jaccard scores of 0.8455, 0.8425, and 0.7341. Compared with the best-performing baseline GAC-Net, DCLA-Net improved Dice, Mcc, and Jaccard by 1.47%, 1.43%, and 2.18%, respectively. Although the performance improvements over some advanced methods are relatively modest, DCLA-Net achieves the highest average values across all three evaluation metrics. Meanwhile, the relatively small standard deviations indicate that the proposed model maintains stable segmentation performance. These results demonstrate that the proposed dual-encoder architecture, together with the multi-scale context-aware fusion module and dual-attention fusion module, can effectively enhance feature representation and improve ectopic pregnancy mass segmentation performance.

**Table 2:** Comparative study on the ectopic pregnancy mass dataset.

Method	Dice	Mcc	Jaccard
TransRUPNet (Jha et al., 2024a)	0.7236 ± 0.0475	0.7200 ± 0.0475	0.5691 ± 0.0592
TransResU-Net (Tomar et al., 2022)	0.7488 ± 0.0513	0.7460 ± 0.0522	0.6012 ± 0.0659
PVTFormer (Jha et al., 2024b)	0.6841 ± 0.0507	0.6794 ± 0.0507	0.5221 ± 0.0577
DoubleU-Net (Jha et al., 2020)	0.7841 ± 0.0403	0.7813 ± 0.0408	0.6466 ± 0.0534
I2U-Net (Dai et al., 2024)	0.8258 ± 0.0362	0.8244 ± 0.0362	0.7049 ± 0.0362
DESENet (Tang et al., 2025)	0.8246 ± 0.0420	0.8222 ± 0.0420	0.7038 ± 0.0613
A-Net (Chen et al., 2023)	0.8104 ± 0.0486	0.8074 ± 0.0486	0.6838 ± 0.0646
LMFR-Net (Zhang et al., 2025b)	0.8022 ± 0.0386	0.8010 ± 0.0386	0.6715 ± 0.0386
GAC-Net (Wu et al., 2025)	0.8308 ± 0.0381	0.8282 ± 0.0381	0.7123 ± 0.0549
DCLA-Net	0.8455 ± 0.0367	0.8425 ± 0.0378	0.7341 ± 0.0552

**Figure 10:**
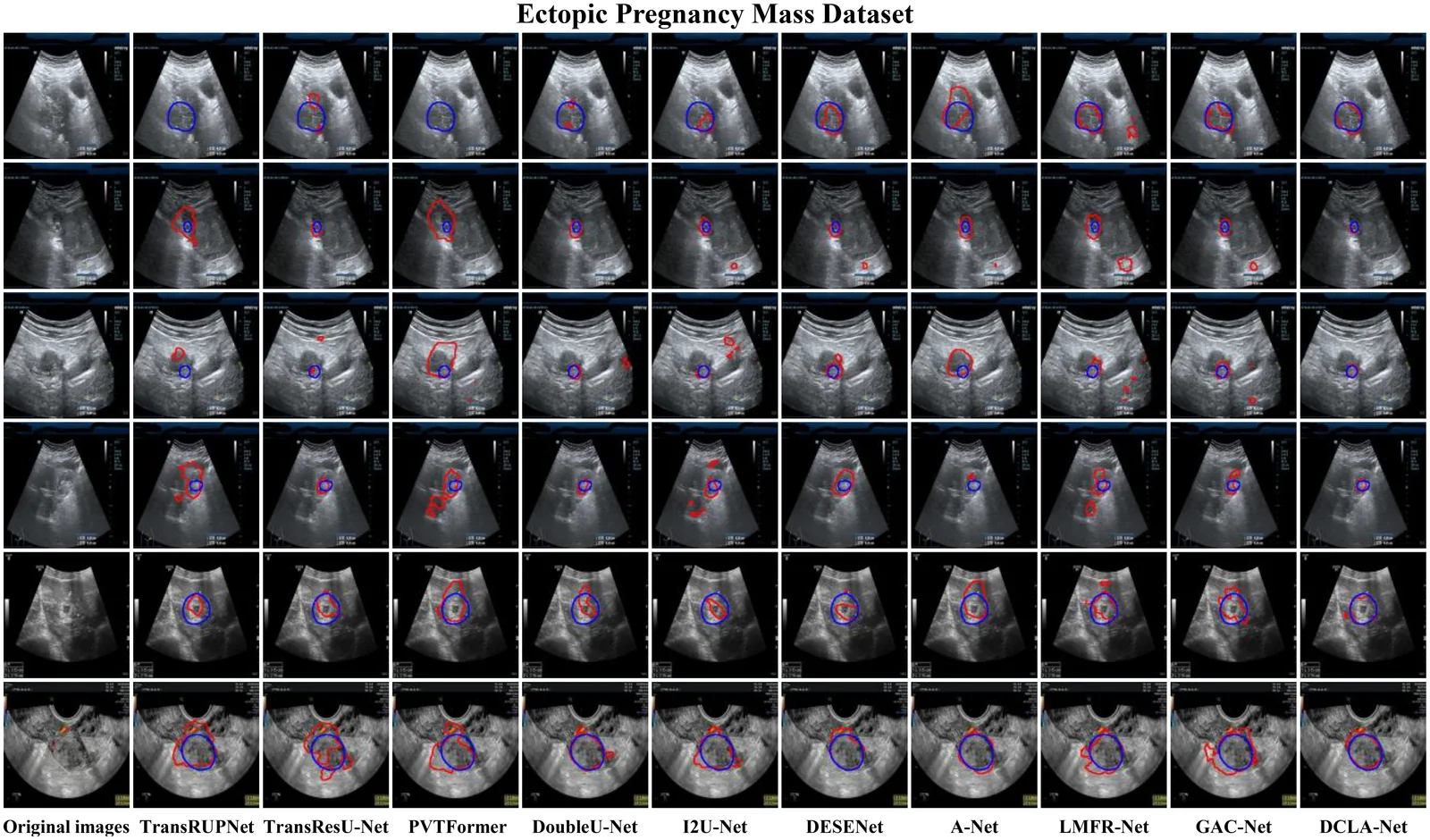
Qualitative comparison of various methods on the ectopic pregnancy mass dataset. The first column shows the original images, while the remaining columns display the results of TransRUPNet, TransResU-Net, PVTFormer, DoubleU-Net, I2U-Net, DESENet, A-Net, LMFR-Net, GAC-Net and DCLA-Net.

As shown in **Figure 10**, the qualitative results further demonstrate the superiority of the proposed DCLA-Net on the Ectopic Pregnancy Mass dataset. Compared with the ground-truth masks, several competing methods produce incomplete or fragmented predictions, especially when the lesion area is small, the boundary is ambiguous, or the surrounding tissues have similar intensity. The Transformer-based methods show unstable segmentation results in some cases, with missed detections or scattered false-positive regions, indicating that relying mainly on global modeling may be insufficient for preserving fine lesion details in ultrasound images. The dual-branch models generally obtain more complete target regions than the Transformer-based methods, but they still suffer from irregular contours and inaccurate localization in challenging samples. The conventional encoder-decoder models show relatively competitive performance, but some predictions remain over-segmented or under-segmented, particularly around blurred boundaries. In contrast, DCLA-Net generates segmentation masks that are more consistent with the annotations in terms of lesion position, shape, and boundary structure. These visual results indicate that the dual-encoder design, MSCAFM, and DAFM work together to improve local detail extraction, global context modeling, and multi-level feature fusion, leading to more accurate and robust segmentation results.

**Table 3** presents the quantitative comparison of DCLA-Net with several representative segmentation methods on the Carotid dataset. The results are reported as mean ± standard deviation to evaluate both segmentation accuracy and performance stability. Compared with the Ectopic Pregnancy Mass dataset, all methods achieve relatively high scores on the Carotid dataset, with Dice values ranging from 0.9496 to 0.9597, indicating that carotid artery segmentation is relatively less challenging under this dataset setting. Among the compared methods, TransRUPNet obtains Dice score of 0.9496, while the other approaches achieve comparable performance, with Dice values exceeding 0.95. In particular, TransResU-Net, A-Net, and GAC-Net achieve Dice scores of 0.9587, 0.9582, and 0.9576, demonstrating their effectiveness for carotid artery segmentation. DCLA-Net achieves the highest numerical results among the evaluated methods, with Dice, Mcc, and Jaccard scores of 0.9597 ± 0.0118, 0.9588 ± 0.0120, and 0.9227 ± 0.0215. Compared with TransResU-Net, which obtains the second-highest Dice score, DCLA-Net improves Dice by 0.10%, while maintaining comparable Mcc and Jaccard performance. Considering the overall high segmentation accuracy of this dataset, the performance differences among advanced methods are relatively small. Nevertheless, the stable results reflected by the standard deviations indicate that DCLA-Net can achieve reliable segmentation performance on carotid ultrasound images. These results suggest that the proposed feature alignment and attention fusion strategies provide additional representation capability under high-quality ultrasound segmentation scenarios.

**Table 3:** Comparative study on the carotid dataset.

Method	Dice	Mcc	Jaccard
TransRUPNet (Jha et al., 2024a)	0.9496 ± 0.0129	0.9486 ± 0.0130	0.9044 ± 0.0130
TransResU-Net (Tomar et al., 2022)	0.9587 ± 0.0150	0.9578 ± 0.0150	0.9211 ± 0.0268
PVTFormer (Jha et al., 2024b)	0.9549 ± 0.0068	0.9539 ± 0.0069	0.9138 ± 0.0124
DoubleU-Net (Jha et al., 2020)	0.9550 ± 0.0149	0.9542 ± 0.0146	0.9143 ± 0.0266
I2U-Net (Dai et al., 2024)	0.9547 ± 0.0141	0.9537 ± 0.0144	0.9137 ± 0.0251
DESENet (Tang et al., 2025)	0.9537 ± 0.0162	0.9527 ± 0.0165	0.9119 ± 0.0286
A-Net (Chen et al., 2023)	0.9582 ± 0.0078	0.9572 ± 0.0079	0.9198 ± 0.0143
LMFR-Net (Zhang et al., 2025b)	0.9536 ± 0.0082	0.9526 ± 0.0084	0.9115 ± 0.0084
GAC-Net (Wu et al., 2025)	0.9576 ± 0.0094	0.9566 ± 0.0095	0.9187 ± 0.0171
DCLA-Net	0.9597 ± 0.0118	0.9588 ± 0.0120	0.9227 ± 0.0215

**Figure 11:**
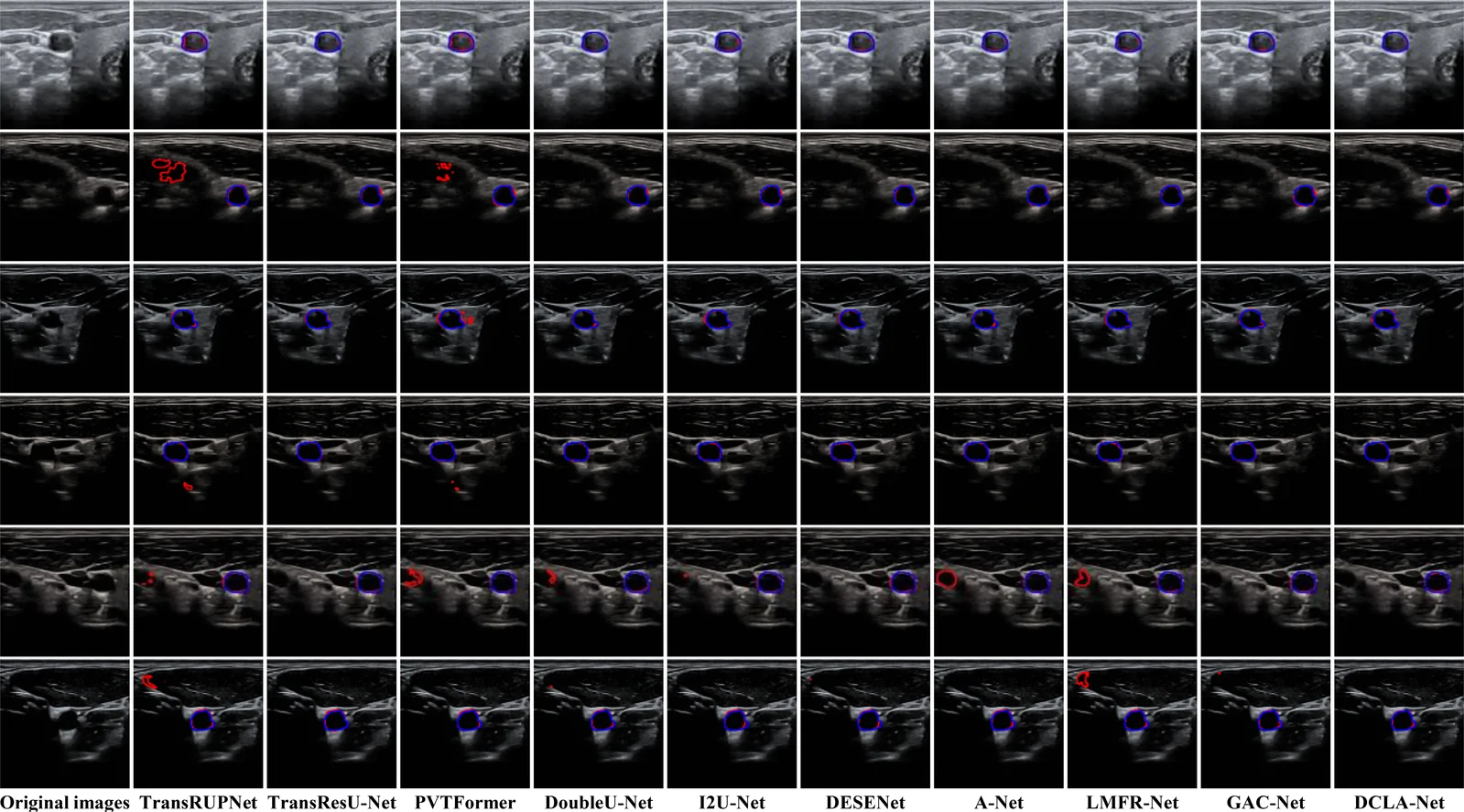
Qualitative comparison of various methods on the carotid dataset. The first column shows the original images, while the remaining columns display the results of TransRUPNet, TransResU-Net, PVTFormer, DoubleU-Net, I2U-Net, DESENet, A-Net, LMFR-Net, GAC-Net and DCLA-Net.

As shown in **Figure 11**, all methods achieve visually satisfactory segmentation results on the Carotid dataset, which is consistent with the high quantitative scores reported in **Table 3**. Most models are able to accurately capture the main structure of the target region, and the predicted masks are generally consistent with the ground-truth annotations. However, closer inspection reveals that several competing methods still exhibit minor deficiencies in certain cases, such as small false-positive regions or partial missing areas, especially when the target boundaries are less distinct or affected by surrounding noise. These issues indicate the presence of occasional over-segmentation and under-segmentation. In contrast, the proposed DCLA-Net produces more precise and stable segmentation results across different samples. The predicted regions show better alignment with the ground truth in terms of shape and boundary, while effectively reducing spurious responses and missed detections. This suggests that DCLA-Net achieves a more balanced and robust performance, further validating the effectiveness of the proposed architecture in handling carotid ultrasound image segmentation.

### 4.6 Computational efficiency

**Table 4** compares the computational efficiency of different methods on the Ectopic Pregnancy Mass and Carotid datasets. Since all methods use identical network architectures on both datasets, the GFLOPs and parameter counts remain unchanged, while only minor differences are observed in FPS. Similar computational efficiency trends are obtained on both datasets. Among the compared models, A-Net has the lowest computational cost, requiring only 0.60 GFLOPs and 0.39M parameters, while LMFR-Net contains the fewest parameters (0.07M) with 3.49 GFLOPs. DESENet and I2U-Net are also lightweight, requiring 3.17 GFLOPs and 1.16M parameters, and 3.56 GFLOPs and 6.76M parameters. In contrast, PVTFormer, GAC-Net, and TransResU-Net require substantially higher computational costs, with 103.95, 81.36, and 41.16 GFLOPs. The proposed DCLA-Net has the highest computational complexity, requiring 177.74 GFLOPs and 108.95M parameters, mainly due to its dual-encoder architecture and feature fusion modules. Nevertheless, DCLA-Net still achieves real-time inference speeds of 111.83 FPS on the Ectopic Pregnancy Mass dataset and 111.51 FPS on the Carotid dataset, which are considerably higher than those of TransResU-Net, I2U-Net, and GAC-Net. Considering that DCLA-Net achieves the best segmentation performance on both datasets, the increased computational cost represents a reasonable trade-off between segmentation accuracy and computational efficiency.

**Table 4:** Computational efficiency on the ectopic pregnancy mass and carotid datasets.

Dataset	Method	GFLOPs	Params(M)	FPS
Ectopic Pregnancy Mass	TransRUPNet (Jha et al., 2024a)	13.91	3.68	211.84
	TransResU-Net (Tomar et al., 2022)	41.16	32.64	17.78
	PVTFormer (Jha et al., 2024b)	103.95	5.08	205.66
	DoubleU-Net (Jha et al., 2020)	53.81	29.29	214.61
	I2U-Net (Dai et al., 2024)	3.56	6.76	42.00
	DESENet (Tang et al., 2025)	3.17	1.16	51.76
	A-Net (Chen et al., 2023)	0.60	0.39	200.14
	LMFR-Net (Zhang et al., 2025b)	3.49	0.07	142.89
	GAC-Net (Wu et al., 2025)	81.36	31.89	13.56
	DCLA-Net	177.74	108.95	111.83
Carotid	TransRUPNet (Jha et al., 2024a)	13.91	3.68	212.98
	TransResU-Net (Tomar et al., 2022)	41.16	32.64	17.63
	PVTFormer (Jha et al., 2024b)	103.95	5.08	207.86
	DoubleU-Net (Jha et al., 2020)	53.81	29.29	212.18
	I2U-Net (Dai et al., 2024)	3.56	6.76	46.16
	DESENet (Tang et al., 2025)	3.17	1.16	154.96
	A-Net (Chen et al., 2023)	0.60	0.39	225.71
	LMFR-Net (Zhang et al., 2025b)	3.49	0.07	167.99
	GAC-Net (Wu et al., 2025)	81.36	31.89	6.81
	DCLA-Net	177.74	108.95	111.51

### 4.7 Optimizer selection

**Table 5** compares the performance of different optimizers on the Ectopic Pregnancy Mass and Carotid datasets. Similar performance trends are observed on both datasets. On the Ectopic Pregnancy Mass dataset, Adam achieves the best performance, with Dice, Mcc, and Jaccard scores of 0.8455, 0.8425, and 0.7341, indicating that it is the most suitable optimizer for training DCLA-Net. AdamW and NAdam obtain slightly lower segmentation performance, while RMSprop performs less favorably. Among all compared optimizers, Rprop yields the lowest performance on the Ectopic Pregnancy Mass dataset. Similar trends are also observed on the Carotid dataset, where Adam again achieves the highest Dice, Mcc, and Jaccard scores of 0.9597, 0.9588, and 0.9227. Overall, these results demonstrate that Adam consistently provides the best optimization performance across both datasets and is therefore adopted for all subsequent experiments.

**Table 5:** Optimizer selection on the ectopic pregnancy mass and carotid datasets.

Dataset	Optimizer	Dice	Mcc	Jaccard
Ectopic Pregnancy Mass	Adam	0.8455	0.8425	0.7341
	AdamW	0.8111	0.8085	0.6850
	NAdam	0.8256	0.8237	0.7042
	RMSprop	0.7956	0.7950	0.6624
	Rprop	0.4315	0.4423	0.2771
Carotid	Adam	0.9597	0.9588	0.9227
	AdamW	0.9564	0.9554	0.9167
	NAdam	0.9567	0.9557	0.9171
	RMSprop	0.9528	0.9517	0.9104
	Rprop	0.9540	0.9530	0.9124

## 5 Conclusion

This study presents DCLA-Net, a dual-encoder cross-level alignment network for ectopic pregnancy mass segmentation in ultrasound images. The framework combines a CNN pathway for detailed local feature extraction with a Transformer pathway for global context modeling, enabling more comprehensive feature representation. In addition, the proposed multi-scale context-aware fusion module facilitates effective interaction across different semantic levels, while the dual-attention fusion module enhances feature refinement by jointly leveraging channel and spatial information. Experimental results on both the Ectopic Pregnancy Mass dataset and the Carotid dataset demonstrate that DCLA-Net consistently outperforms several representative methods across Dice, Mcc, and Jaccard metrics. However, the improved segmentation performance is accompanied by increased computational complexity, mainly due to the introduction of the Transformer modules, MSCAFM, and DAFM. Despite this limitation, the model maintains an inference speed of 111.83 FPS under the experimental setting. Future work will focus on developing lightweight versions of DCLA-Net through module simplification, efficient feature fusion, and model compression, while extending the proposed framework to other medical imaging tasks to further evaluate its generalization ability and practical applicability.

## Data Availability

The raw data supporting the conclusions of this article will be made available by the authors, without undue reservation.
